# Young Women’s Perspectives of Their Adolescent Treatment Programs: A Qualitative Study

**DOI:** 10.3390/ijerph15020373

**Published:** 2018-02-22

**Authors:** Miriam Clark, Rohanna Buchanan, Leslie D. Leve

**Affiliations:** 1 Oregon Social Learning Center, Eugene, OR 97401, USA; miriamc@oslc.org; 2 College of Education, University of Oregon, Eugene, OR 97403, USA; leve@uoregon.edu

**Keywords:** teenage pregnancies, unwanted pregnancies, child abuse, substance use, health risking sexual behavior, mental health, services for adolescents

## Abstract

The perspectives of at-risk adolescent clients can play an important role in informing treatment services. The current study examines qualitative interview data from 15 young women with histories of maltreatment. Using a semi-structured qualitative interview approach, we asked the women to think retrospectively about their treatment experiences as adolescent girls. Results highlight the need for providing adolescent girls with reliable and practical information about risky sexual behavior and drug use from relatable and trustworthy helping professionals. We discuss strategies for developing and maintaining trust and delivering specific content.

## 1. Introduction

Childhood maltreatment is associated with an elevated risk of adolescent drug use and risky sexual behavior [1]. Both adolescents and adults with histories of maltreatment are more likely to have sex while drinking or using drugs, which often results in unprotected casual sex—leading to sexually transmitted infections and unwanted pregnancies [2,3,4]. Additionally, adults with histories of childhood trauma are more likely to exchange sex for drugs or money [5,6], be arrested for prostitution, have unprotected sex with casual partners [1], and have a high sensitivity to social rejection which leads to more risky sexual encounters [7].

Several evidence-based treatment programs have demonstrated effectiveness in reducing the probability of adolescents with histories of maltreatment engaging in drug use and risky sexual behavior (see Multisystemic Therapy, Treatment Foster Care Oregon, Functional Family Therapy, and Trauma-Focused Cognitive–Behavioral Therapy) [8]. However, one area that has received little attention in developing and refining treatment programs is the importance of directly soliciting clients’ opinions and input [9]. Client input can provide valuable insight into the client/clinician or client/intervention relationship. In fact, Hodgetts and Wright [10] argue that researchers cannot fully understand the impact of treatment without learning about and incorporating the clients’ experiences and perspectives.

Numerous studies have explored adolescents’ views of their interactions with helping professionals including mental health therapists, mentors, doctors, nurses, or other treatment providers [10,11,12,13,14,15,16]. However, these studies often lack specific information about how client-clinician relationships are developed and built over time. For example, in a review of 54 qualitative studies examining adolescents’ views on their relationships with helping professionals, Freake and colleagues [12] identified 12 important themes that regularly emerge in qualitative data. The 12 themes include confidentiality, giving advice, listening, kindness, trustworthiness, being qualified, being non-patronizing, being non-judgmental, being easy to talk to, consistency, being treated as an individual, and—for medical issues specifically—that adolescent girls prefer a female doctor. Although these themes have been examined broadly in prior research, Freake and colleagues identified a need for researchers to look deeper into these factors and examine how adolescents’ perceptions about treatment develop over time. Specifically, Freake and colleagues report that prior research shows that helping professionals often lack an understanding about how clients want professionals to explain things, what being “easy to talk to” means, and how trust can be developed and sustained. 

### The Current Study

One strategy to gain insight into how the adolescent client/clinician relationship changes over time is to ask young adults how they viewed the treatment programs they attended and to solicit their suggestions for future programs. The retrospective nature of this type of inquiry brings added insight that is not possible when questions are asked during treatment [17]. Retrospective inquiry is a critical tool in the current study because research has shown that impulsivity and sensation seeking behaviors are at their peak during adolescence, that adolescents often feel invincible, that futuristic thinking is uncommon (adolescents live in the moment), and that the adolescent brain continues to develop well into the mid-20’s [18,19]. Because of these features of human development, most adolescents are not able to accurately perspective-take and reflect on their own current behaviors and associated risks and needs. Waiting until individuals are in their 20’s, and then asking them about their teen years, means that most individuals have developed a greater capacity to reflect on their choices and what was/was not helpful during adolescence and decisions they wished they had made, yet they are still close enough to the period of adolescence to have a deep understanding of what it is like to be a teen in today’s world. Following this approach, we conducted 15 semi-structured, open-ended qualitative interviews with young women with a history of maltreatment and placement in out-of-home care. During the interviews, we asked the women to reflect on their experiences with treatment programs. Three research questions guided the qualitative interviews and analyses:

1. What types of treatment services did young women with histories of maltreatment experience as adolescents?
2. What feedback do young women with histories of maltreatment have that relates to both positive and negative characteristics of helping professionals and treatment programs for adolescent girls with histories of maltreatment?
3. What feedback do young women with histories of maltreatment have to inform treatment program content and delivery related to drug use, risky sexual activity, and partner choice for adolescent girls with histories of maltreatment?

## 2. Materials and Methods

### 2.1. Participants

Young women (n = 15) with maltreatment histories who had participated in treatment programs as adolescents were recruited for the current study. The women were identified from two completed, randomized, controlled trials in which they had participated during adolescence [20,21] and from a local community mental health center. Recruitment included an initial telephone call to determine interest and eligibility for participation followed by an in-person, individually administered IRB-approved informed consent procedure. The IRB protocol number is 10312013.040 from the University of Oregon. Interview content was examined after each interview and we determined that we reached sufficient data saturation [22] with the sample of 15 women. All recruitment and interview activity took place between November of 2013 and April of 2014.

### 2.2. Measures

**Interview guide.** We used an individually administered, semi-structured, and open-ended qualitative interview format and conducted one interview with each participant. We designed the questions in the interview guide to prompt respondents to think retrospectively about treatment programs they attended during their adolescent years and to solicit information about the topics most important to them. Asking participants retrospectively about the programs in which they had participated allowed them to think critically about how these programs might have influenced their lives. The treatment-related questions in the interview guide were general in order to allow respondents to formulate their own unbiased responses. Questions asked participants to think retrospectively about their treatment experiences as adolescents and explain the treatment elements that were helpful or not helpful for them. Example questions included: “What parts of the program did you find helpful?”, “What was helpful about those parts?”, “Were there any parts of the program you did not think were helpful?”, “Why did you not think they were helpful?”. Interviewers were trained in techniques to elicit information from participants, remain neutral to a range of responses, and move the discussion through the semi-structured format.

**Demographic survey.** Each participant completed a demographic survey prior to the interview. The demographic survey included items related to the participants’ age, race/ethnicity, current relationship status, and education. 

### 2.3. Analysis

Interviews were conducted by two graduate research assistants. Both interviewers were trained in qualitative interview techniques which allowed participants to feel comfortable to express their opinions naturally while moving through the semi-structured interview guide. All interviews were audio recorded and transcribed verbatim. The interviewers then reviewed each transcript for accuracy. We assigned participants, other individuals, and treatment programs identified in the transcripts an identification number prior to analysis to protect confidentiality and reduce subjectivity in the analyses [22]. After reviewing all of the interviews, the authors met and discussed broad themes identified across all transcripts. The first author coded all of the interview transcripts. Using NVIVO software [23], she completed an initial coding of the broad themes for each interview. Next, a codebook emerged as the first author coded sections of text (ranging in size from short phrases to long discussions) by hand into more specific emergent sub-themes, then compared the coded content across each interview. The coder grouped codes into themes and subthemes using the method of constant comparison [24]. All authors met to thoroughly review the first author’s analysis, discuss findings related to the broad themes and sub-themes, and to agree on representative quotes.

### 2.4. Reflexivity Statement

The first author and coder for the study is a research assistant with a MS in sociology. She is trained in qualitative research methodology including developing interview questions, conducting interviews, and coding and analyzing qualitative data. She has 9 years of experience conducting qualitative and quantitative interviews and assessments in English and Spanish with children, adolescents, and parents from racially and economically diverse populations. She has assisted with the development of research protocols and procedures for multiple research studies with children and adolescents. The second author has a Ph.D. in school psychology. She is clinically trained in behavioral treatment models and has been involved in the mental health treatment of children and adolescents for 18 years. Her experience includes serving as a clinician, clinical supervisor, and principal investigator on a number of research studies with children and adolescents involved with juvenile justice, child welfare, and special education systems. She has significant experience developing and implementing treatments for adolescent girls. The third author has a Ph.D. in developmental psychology and has been studying both typical and atypical child and adolescent development for the past 20 years. For the past 15 years, she has been part of a research team evaluating developmental pathways and intervention outcomes for girls with child welfare and juvenile justice involvement. Her roles have included theoretical conceptualization, assessment development, data analysis, and manuscript development (she is not clinically trained and does not serve clients).

## 3. Results

### 3.1. Quantitative Results: Participants and Interviews

The young women in the current study were 18–24 years of age (M = 20.93, SD = 2.09) at the time of the interview. Participants identified as White or Caucasian (60%), American Indian/Alaska Native (7%), or more than one race (33%). Overall, 20% of the sample identified as Hispanic/Latino. Participants reported that they were married (n = 1), living with a partner and unmarried (n = 6), or were dating or seeing someone but not living together or married (n = 8). Participants reported a range of education attainment including having completed the 8th grade (n = 1), having completed some high school (n = 4), having received a GED (n = 3), having received a high school diploma (n = 5), and having completed some college (n = 2). The sample was generally unemployed and low income, with only four women employed for pay and a median income range of $5000–$9999.

Interviews were scheduled for two hours. The average interview length was 102 min (range = 66–136 min).

### 3.2. Broad Themes from the Qualitative Interviews

When talking about their prior treatment experiences, the women told highly detailed and lengthy stories that were sometimes directly related to the interview questions and sometimes more tangential to the focus of the question. The stories focused on a range of programs including sex education classes in school, day-treatment programs, treatment foster care, independent living programs, and residential treatment programs. Three broad themes emerged from the interviews: positive experiences from past programs, negative experiences from past programs, and suggestions for developing effective treatment programs. We grouped subthemes within these broad themes.

### 3.3. Positive Experiences from Past Programs

All 15 women identified positive past experiences in treatment programs, sex education, and/or other means of gaining information about risk and prevention related to sexual behavior and drug use. Participants’ positive experiences were grouped into five sub-themes: (a) traits of the helping professional, (b) knowledge gained, (c) positive social activities, (d) social support from the helping professional and (e) specific resources found helpful. 

**Traits of the helping professional.** Fourteen participants discussed positive qualities they noticed in the helping professionals they had interacted with as adolescents. The women identified a range of positive qualities regarding the helping professional’s approach including being non-judgmental, being understanding, being honest, keeping information confidential, being interested in the adolescent, having similar past experiences as the adolescent, and rewarding positive behavior. The women also identified positive qualities that made the helping professional easier to connect with including being down to earth, funny, young, a friend, kind, outgoing, female, easygoing, and interested in specific activities such as art or sports. One participant summed up many of these characteristics when she discussed her favorite counselor fondly:

“My counselor…was the epitome of an overachieving counselor. She was absolutely amazing. She was really young…She dressed like a teenager, and she went through a lot of traumatic events herself growing up. So she not only did she, like, sympathize, she empathized. She went through it. So being able to talk to someone that had already had experience in the matters that I was going through, that really helped…She was like a big sister, and that’s what I think teen girls need if they don’t have an older sister or something they can look up to.”

Another participant highlighted the importance of a non-judgmental and understanding approach, “Cuz she um she knew about everything that I had done but she didn’t … criticize me for it or, or made me feel like I was being judged. She was just always kind of um uh, understanding I guess”.

**Knowledge gained.** Twelve participants identified specific things they remembered learning that they said have helped them. Topics they identified included sex education, coping skills, values such as patience and positivity, and the negative effects of drugs. One of the women highlighted that she had learned specific coping skills, saying, “They went into great length with coping skills. And really great ways to take my anger out in a positive manner: Crocheting, knitting, painting”.

**Positive social activities.** Nine participants identified social and active aspects of programs as valuable. Most of the social experiences discussed by the young women happened with a mentor in a therapeutic program, though some occurred at school or with other helping professionals (e.g., counselor, therapist). Activities mentioned included art, hiking, work experience, sports, hanging out, movies, music, and school activities. The women talked about the importance of having fun and doing exciting things to replace the desire for drugs and alcohol. One of the young women stated, “That’s what’s good about the BSS [behavior support specialist]. Cuz they like took you out of the situation and taught you, ‘you know, we can go rollerblading, instead of going to smoke crack.’” Another woman identified that she had a history of substance use as an adolescent but she was sober when she engaged in social activities, “I did, uh, youth group, campus life is what it was called. And I did basketball; I did volleyball”. 

**Social support from the helping professional.** The opportunity to talk to or spend time with a helping professional was something ten participants said they enjoyed and found helpful. While most of the women simply said that it was nice to have someone to talk to, one participant talked about the help she received saying, “Counseling really helped me. Like just being able to talk to someone and them, like, not judge me and to be able to talk back to me, is like, I dunno, I feel like an equal when I talk to a counselor, a lot of the time.” Another participant described the support she received from her mentor in more detail,

“It was just nice to have somebody to kind of hang out with and talk to that wasn’t my grandma because I was living with her at the time. And um, yeah, it was just it was nice having somebody to go to. Definitely just hang out with…it was weird how it helped out…it kind of boost my self-esteem a little bit… I think how to kind of to cope a little more with not being able to see my family that much. And being able to see her [mentor] kind of took my mind off of, kind of, the sad parts of my life back then.”

**Specific resources found helpful.** Nine participants listed specific resources they found helpful from various treatment and education programs. These resources included medication; food; shelter; birth control; help with resumes; help obtaining a driver’s license; financial help; help with college tuition; classes in budgeting, cooking, and shopping; STI testing; support groups for LGBT adolescents; clothing; bus passes; washing machines; and a place to hang out for adolescents. One participant expressed her appreciation for these resources, “I was in [an] independent living program…And we would…do resumes and, um, talk about, like, getting food stamps and drivers licenses…They gave me $600 a month. They helped out with college. That was an awesome program”.

### 3.4. Negative Experiences from Past Programs

All 15 women talked about past negative experiences in treatment programs or sex education; these were grouped into seven sub-themes: (a) lack of information about sex and drugs, (b) negative qualities of the helping professional, (c) adolescent did not participate in or appreciate program, (d) adolescent did not like characteristics of the facility or program, (e) high turnover rate of helping professionals, and (f) worries about confidentiality.

**Lack of information about sex and drugs**. One sub-theme that emerged in 13 of the interviews was that adolescents lack information about safe sex practices and the risks of drug use. The women emphasized that prior treatment and education programs had not provided sufficient practical information for them to make healthy and safe choices. The women talked about how adolescents’ lack of such information is a result of misinformation, not receiving information in the first place, or not believing the information they did receive. For example, one respondent said, “definitely some people aren’t aware of how some sexual transmitted diseases [STIs] aren’t protected from things like condoms, and they don’t think about things like blowjobs and things like that and how you could receive it [an STI] orally.” Another participant described that some adolescents do not know where to obtain information about safe sex, saying:

“I just feel that and with their [adolescents] age being so young now I think that they’re embarrassed to talk to their parents [about sex]. I think that they, um, just they don’t know what their parents will say. They don’t know what their friends will say. Um, sometimes, they don’t … have that much information…, but they don’t know where to start…”

**Negative qualities of the helping professional.** Ten participants discussed negative qualities they remembered in their relationship with their helping professional. Negative qualities in the relationships varied in nature, but all contributed to an environment where the adolescents had difficulty building rapport. Participants mentioned things such as feeling judged unfairly, feeling a lack of trust, feeling that meetings with the helping professional were not frequent enough, and feeling that the helping professional was hard to relate to due to an age difference. One participant described her difficulty building rapport with counselors this way, “They just didn’t, they seemed so unattached to the situation. …they had no idea they didn’t even really wanna be there it seemed like with some of ‘em. And they were just doing it kinda for a job…” Another participant discussed how she felt that her helping professional did not trust her,

“…the things I told them about my parents and stuff, they didn’t believe any of it, pretty much. That, that was a pretty big deal, cuz my dad was very, my dad was abusive and stuff and I had a lotta issues at home and nobody heard that, so and they acted like I’m the problem.”

Another participant explained, when asked if she felt comfortable talking to her mentor about sex, how she felt that she would have been judged had she gone into too much detail, “I felt comfortable talking to her about the idea of sex, but not like actual. Cuz she’s an adult and sometimes it’s just awkward because you feel like they’re going to judge you”. 

**Adolescent did not participate in or appreciate program.** Ten participants reported feeling that treatment was not a good fit for them as an adolescent because of their own feelings toward the program. They talked about how they refused to participate (by either not attending or remaining silent in treatment), not liking the program, feeling that they were forced into treatment, or feeling that they were not ready for treatment. For example, one participant said, “Um, it, um [sighs] well, I wasn’t ready for services. Um, that is the main reason why it didn’t work, was because I wasn’t ready to quit [using drugs].” Another participant described her negative experience in a residential program,

“I did not like being around a bunch of people that I didn’t want to be around, that I was forced to be around. Like the girls in the programs that had issues and stuff. It made it harder and what else? I didn’t like not having my own stuff and being told what to do every day.”

Half of these women who expressed negative feelings toward the program also expressed regret that they had had these negative feelings. For example, one woman expressed her regret for not attending, saying, “I wish I would have gone more [to therapy]… I had to actually go down there and I just never [did,] I would rather hang out with my friends.” Another participant said, “I wished I had been more open with him [her therapist]. I probably would’ve gotten better treatment”.

**Adolescent did not like characteristics of the facility or program**. Eight of the women mentioned specific things they did not like about the facility or the program. Six women said they did not like being in a group treatment program and gave examples related to the structure, the classes, the lack of funding, feeling forced to be there, or the location of services. One of the women highlighted the lack of funding by saying, “I feel that [the program] isn’t being given enough money and I know that just this whole economy sucks, but they are doing a lot for us [clients], and um, they just don’t have the adequate supplies they need. I mean, they don’t even have a stove in their kitchen.” Three of the women said they did not like being in therapy in general because they felt that they did not have enough say in the therapy received, their family did not come to family therapy, or they did not like being forced to open up to a stranger (e.g., new therapist). Two women mentioned that they felt like they were over medicated. One of these women described her frustration with the treatment program by saying, “I just didn’t appreciate the fact that they tried to get me to take things [medication] that I didn’t need”.

**High turnover rate of helping professionals.** Five respondents discussed the high turnover rate of the helping professional (e.g., mentor, worker, therapist) they had been assigned as an adolescent. Some of the women talked about their experience with therapist turnover being due to the high turnover at the agency, while others talked about how they moved often and had to change helping professionals as a result of the move. One woman described her experience with turnover this way:

“I wished that they had made a contract with ILP [independent living program] workers that they had to work, they couldn’t just quit. They had to work for a set period of time. …I went through about four or five ILP workers because they found different jobs, or they just randomly decided to switch their caseload, and then they wouldn’t really tell me about it or anything, and it just kind of happened.”

Regardless of the reason for the turnover, the women reported that the high turnover rate made it difficult to build rapport and open up to a new helping professional. The women talked about how they felt they needed to be more guarded over time as they experienced more therapist turnover and had difficulty putting trust in someone that might leave. Another participant talked about moving and her experience with turnover, saying:

“…once I turned …fourteen I was like, “I’m not doing counseling anymore.” And they’d make me go and I’d just sit there. And I wouldn’t say nothing, cuz I was just like, I’m, I’m tired of connecting with somebody, and like, everything switches every time I move, you know what I mean? It’s pointless.”

**Worries about confidentiality.** Three participants said that they had not wanted to open up to a helping professional out of fear the helping professional would break confidentiality. The way the women talked about their concerns related to confidentiality suggested that, as adolescents, they were unclear about mandatory reporting rules. One participant shared her story like this:

“I didn’t really feel comfortable really talking about drugs to a counselor cuz they say in the beginning you know, this is confidential unless we feel like you’re harming yourself or others. And you kinda are when you do those. So, like you like feel like you’re—you can’t really say anything at all.”

Two other participants said that their counselor did break confidentiality and one explained her story this way:

“I did not like the fact that every time we talked, she would, you know, she would write down everything I said. And then she’d go off and show it to my parents. I don’t like the breaking of, you know, trust, and confidentiality. I hate that crap.”

### 3.5. Ideas for Developing Effective Treatment Programs for Adolescent Girls with Maltreatment Histories

In addition to questions about their prior intervention experiences, we asked the women about their ideas for effective treatment programs. We asked participants what an ideal program would look like for adolescent girls related to helping professionals and program content.

**Characteristics of the ideal helping professional.** When asked who should deliver specific types of programs or services, participants identified qualities related to age, gender, experience, and personality. These characteristics seemed indicative of the desire to have a helping professional to whom they could relate. For example, one participant said,

“Definitely having someone close to age or the same age ... someone who’s just close to the same personality sometimes is very helpful or like interested in the same activities and uh, yeah, I think that would be really helpful. Because I know the girl I was seeing she was into art and music and that was my big thing and it was nice being able to talk about it or do some art projects.”

*Age.* Nine of the women mentioned the importance of having a young program facilitator, mentor, or therapist. Their definitions of young varied slightly, but all mentioned ages that ranged in the 20s and 30s. When discussing age of helping professionals, one participant said, “I didn’t want someone to act motherly towards me.” Another participant expanded on the same idea when she said,

“Having older people can sometimes make some girls feel judged because, um, that person grew up a little bit different than how girls are growing up now and it might make it, it might make them feel like they are talking to their parents more than talking to a friend. So someone just younger.”

The other six participants did not identify age as an important characteristic of the treatment personnel.

*Gender.* Seven participants said that the gender of the treatment personnel was important. Six out of these seven said that they preferred female mentors or program facilitators. One of those six said that although she preferred a female mentor, there should be a mix of genders for facilitators of treatment groups. Another one of those six said that she would prefer either a woman or a gay male. One participant said that she felt sex education would best be taught by someone of the opposite gender. Another participant said that both sexes should teach adolescents about sex. The remaining seven participants did not list gender as an important characteristic of mentors, sex education teachers, or other helping professionals. 

*Experience.* Eight of the respondents discussed the importance of having someone who has “been through it.” They wanted helping professionals who had experience with drugs, risky sexual behavior, or had experienced traumatic events. One participant described it this way:

“…it’s hard to tell someone that you know where they’re coming from when you’ve never dealt with anything like that before. I know that had I had someone who had actually been through stuff that I had been through, maybe not everything, maybe just one or two things, or maybe just one, I think that would have helped me realize, cuz they know and they’ve, you know, at that point what I would say is they’ve survived. You know, they’ve gotten out. And they, they can do it, obviously I can too.”

Another participant put it this way: “Yeah, personal experience. Um somebody who can relate easily and um, just kind of give, give some sort of hope you know to them whoever they are. Wherever they’re at in their stage, you know, of life.” A different participant explained why having someone who lacked such experience was not helpful:

“Cuz if you’ve never done drugs, you can’t sit there and be like, ‘Do not use drugs, they’re really bad for you,’ because that’s all you hear. …some girl comes up and says, ‘Well why are they really bad? How do they taste? You know, what do they smell like when you’re around them?’ … Then you’re gonna be like, ‘I don’t know.’

*Personality traits*. The women mentioned a variety of personality traits that they found important for treatment personnel. These included things such as being happy, a good friend, committed, nice, logical, relatable, persistent, mature, comfortable, knowledgeable, fun, understandable, helpful, able to listen, fun, honest, and positive. By far the most common theme that came up (in 13 of the interviews) was how much adolescents need to feel understood and accepted. They wanted someone who is understanding, non-judgmental, or open-minded. One participant put it this way, “…what you could do is just uh, make ‘em feel like you’re on their side, I guess with everything and that you’re doing it to help them.” Another participant furthered this idea when she said, “non-judgmental person. Ideal…. Somebody who’s not a cop. [laughs], I mean nothing against cops, officers, policemen. Um, you know but not someone who’s going to make them feel like threatened or make them feel like they’re going to be [threatened]”.

**Ideal program content.** When asked about ideal treatment program content, participants shared ideas that included both information and skills. Themes included consequences of risky sexual behavior and drug use, skills to talk with partners about sex and/or drugs, resources, use of protection during sex, and that it is okay to abstain from sex and/or drugs. 

*Potential consequences of risky sexual behavior and drug use.* Thirteen of the 15 women mentioned the importance of teaching about risks involved with sex and drugs and how engaging in risky behavior can impact their current and future lives. Specifically, the women highlighted potential consequences related to risky sexual behavior and drug use individually, as well as those associated with drug use combined with risky sexual behavior.

Eleven of the women talked about the importance of teaching about potential consequences associated with risky sexual behavior. The specific risks the women identified were physical, social, and emotional. Physical risks included STI transmission (e.g., transmission via oral sex, potential of STI transmission with unprotected sex), pregnancy (e.g., sore breasts, morning sickness, cravings, labor, mood swings), and sexual assault. When the women talked about social risks, they emphasized how potential male partners might not want female partners who had been sexually promiscuous. The emotional risks they highlighted included experiences where male and female partners did not have the same feelings about the sexual relationship. Their stories included situations where the male partners lied to get sex, or where the female was in love but the male was just interested in sex. One participant talked about gender differences she had noticed in adolescent views of sex, “Cuz for girls it’s emotional bonding… for boys it’s just getting in your pants. That’s what I’ve experienced anyway. It’s really sad but it’s the truth.” Another participant warned, “Just because he says he cares about you before doesn’t mean that he still will afterwards.” The participants also warned that pressure from a partner to have sex means you are not being respected by the partner.

Many of the women expressed how important it was for treatment programs to provide in-depth information about these potential consequences. One participant explained how to talk about the realities of pregnancy,

“Like telling them the pros and cons of getting pregnant and having a baby. And tell them that it’s worth waiting. It is hard to do school and raise a kid, but I think if they really just talk about some of the unpleasant stuff about being pregnant that a lot of the girls would really be okay to wait. Yeah, once I got pregnant there was a lot of stuff I did not know about. I took health and family’s class and I didn’t know that you could get hemorrhoids. And you’re just very uncomfortable especially once you get bigger and your mood swings really do go up and down.”

Six women talked about teaching adolescent girls about the potential consequences associated with drug use. The specific risks identified focused on incarceration (detention or jail), social effects (e.g., intoxicated friends might steal your things), physical effects, and safety. When talking about the physical effects of drug use, the women highlighted both long- and short-term health effects (e.g., damage to the heart, looking older than their age).

Most respondents suggested that explaining the risks associated with drug use would deter adolescent girls from using. For example, one woman said, “Show them the really bad adverse side effects of using any amount of, you know, alcohol, drugs, hallucinogenics.” Another participant said,

“I mean, let them know the risks, really. I mean, if half of the girls knew what some of the drugs could do to your body, I think a lot of people wouldn’t use ’em. What it can do to you. I mean it can completely change you and you can become someone who you never thought you would. …if I could tell people, you know, a life story and have them not use… I just, it’s not worth it.”

Some participants gave suggestions for how to be safe when using drugs such as obtaining drugs from a trusted source to avoid impurities/contamination and using in a private space because it is easier for people to take advantage of you when you are in a public place. One woman said,

“Like just don’t just do it from some stranger or, you know, somebody you don’t know that well. …you have to be in a really safe environment. Um, my advice … have like someone who at least is like somewhat sober or all the way sober. To, like, you know like if something did happen that they could actually take care of the situation instead of being oblivious to everything.”

When asked what advice they would give to adolescent girls, four women talked about teaching the risks associated with drug use combined with sexual activity and relationships. The women’s stories illustrated three specific types of risks: (1) risks associated with men who use drugs, (2) risk of judgement being impaired when under the influence of drugs/alcohol during sex, and (3) drugs having consequences that affect your relationships—both current relationships and future relationships. For example, one participant said she would, “Recommend the girl get away from that situation and that area because it’s usually the area you know, her sense of home that’s keeping her there in the first place. It’s not just the boyfriend and girlfriend.”

*Skills to talk with partner about sex and/or drugs.* Ten women discussed the importance of teaching adolescents how to talk to their partner about sex and/or drugs. One woman talked about how programs should teach adolescent girls skills to talk to partners so the men listen and understand them, saying, “…like figure out how guys wanna hear what they’re [girls] trying to say and teach ’em how to say it.” Another participant explained what programs can teach adolescent girls to say to partners this way,

“Tell the girl, “Hey, when you, if you’re gonna decide to do something with somebody, you guys should sit down and talk about it first. You know, like, ‘Do you want to use protection?...’ Talk about what it is you’re gonna do before you do it. You know, try to get the guy to, you know, be more sensitive to your needs, you know and don’t just hop into it. Be like, ‘Hey wait, can we talk about this first?’ You know. ‘Maybe can we wait?’”

*Resources.* Eleven women suggested teaching adolescents how to find resources (e.g., condoms, medical services, social support, housing). Four participants talked about the need to connect adolescents to resources for STI and pregnancy prevention, and three talked specifically about getting information and medical care from a community health clinic (“go to Planned Parenthood… They’re a really good resource with condoms or Plan B or whatever you need”). Three participants said it was important to teach adolescents to utilize their parents or other trusted adults as resources about healthy and safe sex practices (“Maybe to encourage ’em maybe to talk to their parents about it [sex]”). Related to housing, one participant talked about the need for adolescents to learn how to find safe housing. Her experience illustrated that adolescent girls often stay in unhealthy relationships because they are living with their partner. She emphasized the need for adolescents to find alternative housing in order to escape unhealthy or unsafe relationships (“people should always have … a little piece of paper … that they always have access to that has certain phone numbers on there so they can always…call for help”). Another participant talked about the connection between sex and access to resources such as housing, food, and security. This participant wanted programs to teach adolescents how to ask for help because there are alternatives to using sex to access resources. She suggested “…going to your nearest DHS office and asking for help. Explaining to them your situation or going to [facility A] or [facility B] and explaining to them. Cuz … there’s a lot of resources to help them.”

*Use of protection during sex.* Eleven participants said that adolescents need to be taught to think about and use protection during sex. Specific examples included teaching adolescent girls to think about the importance of safe sex practices; and to obtain, use, and store different types of birth control/STI prevention (e.g., “condoms,” “Plan B”). For example, one woman explained why adolescents need to know how to correctly use a condom,

“… like a condom, with people is like, when are you gonna stop? And then you have to put it on, you know? And if a girl doesn’t know how to put it on, then she’s not gonna try right in front of the person. So definitely learning how to put a condom on. Learning you know how to keep yourself clean.”

*It is okay to abstain from sex and/or drugs.* Eleven participants said that adolescents need to learn to choose for themselves when to use drugs or have sex. Related to drugs, one participant said, “Just teach them as best you can about peer pressure.” She went on to say, “And that your friends are either gonna be your friends or their not really gonna, or their not your friends at all.” Related to sex, the main sentiment expressed by the women was that adolescent girls should not feel pressure to have sex if they are in a relationship or their friends are sexually active. The women wanted adolescent girls to learn that they should not feel shame for not being ready to have sex, to not worry about being mocked for abstaining from sex, and “that it shouldn’t be a race” to lose their virginity. One participant talked about “…not doing it until they’re ready and not feeling ashamed if they realize they weren’t ready.” Another participant expressed her regret that she had had sex so young and advocated teaching adolescent girls to make their own decisions about sex, saying,

“Try and go to abstinence. Yeah…sex at an early age, I, I regret it. Um, I, that’s just how it is. You do it and then you go, ‘wow that wasn’t great, I regret it.’ And you will regret it for the rest of your life, or most of it…oh I think it might help out a lot of girls. Cuz they’ll be like, ‘okay, I don’t have to do it if I don’t want to.’ Um, it’ll teach girls more about … having an opinion and having their own free will.”

Throughout the interviews, the women emphasized that adolescent girls should learn that it is worth waiting to have sex until they are ready.

## 4. Discussion

The women in the sample were highly engaged in the interviews as evidenced by the detail of their responses and length of the interviews. Participants provided lengthy, in-depth insights into their prior treatment experiences and suggestions for improving treatment programs and relationships with helping professionals. Though some of the feedback is less feasible to implement (e.g., hiring young, skilled therapists who have recovered from prior drug abuse), much of the women’s feedback can be readily implemented and is consistent with recommendations from the treatment literature. 

This study adds to the current body of literature in two important ways. First, asking young adults retrospectively about their treatment programs provides insight into how their perceptions related to program effectiveness have developed over time. Second, asking women with histories of maltreatment about their treatment experiences provides insight into an at-risk population. While our participants identified desired characteristics of helping professionals that are consistent with findings in prior studies [12], this study further develops those themes and helps to fill the gaps in the extant literature by examining how helping professionals can develop and maintain relationships over time. 

### 4.1. Implications for Practice

The comments reflected by the women in the sample illustrated the juxtaposition between adolescents as thrill seekers while simultaneously seeking stability and consistency. Their stories repeatedly demonstrated a desire for stability (represented by being taken care of by male partners and frustration with therapist turnover) contrasted with serious thrill-seeking behavior (represented by things that are potentially harmful e.g., risky sex and/or drug use; or things that are prosocial e.g., sports, art, clubs). This juxtaposition is consistent with literature showing that adolescents are both thrill seekers [18,19] and positively respond to structure and stability [25].

Participants emphasized the importance of giving adolescent girls information. They repeatedly made suggestions about what treatment programs should tell adolescent girls about the risks of sex and drugs and how to access community resources. To a lesser extent, their stories highlight the importance of teaching safety and refusal skills or skills for accessing family or community support. The current literature shows that information, skills, and social support are necessary to reduce risk and promote healthy behavior in adolescents. For example, the Centers for Disease Control and Prevention (CDC) identified that effective HIV/STD [26,27] prevention programs for adolescents include skill-building components and support from family in addition to knowledge gained. Additionally, because there has been an increased use of technology by adolescents, smart phone applications or other digital methods might be an effective strategy for improving access to information. Current research suggests that there is a lack of an effective strategy to provide such information using technology [28] and that even advanced high school students struggle to judge the quality and accuracy of information they find online [29].

The women’s stories indirectly highlight the importance of having agency over one’s body. Their experiences show that they felt like they did not exert control over their own sexual behavior and/or substance use as adolescents, which was directly linked to not having agency over their lives in a broader sense. The women’s suggestions for treatment programs largely stemmed from not wanting other maltreated adolescents to be taken advantage of (the way they were) and therefore wanting to provide adolescents with knowledge and skills to make healthy choices.

**Characteristics of the helping professional.** Consistent with findings from prior research [12], the women’s reports of positive experiences with helping professionals were linked to trusting relationships with providers they could relate to and who had empathy for them and their experiences. The women highlighted qualities such as being non-judgmental, understanding, honest, and maintaining confidentiality as positive qualities of helping professionals. They found the support from helping professionals from their prior treatment programs to be helpful and enjoyed spending time talking and doing activities with them. 

Many of the participants described their ideal helping professional as a young female who could relate to teen clients because she had overcome adversity in her own life (e.g., childhood maltreatment, drug use). Their stories showed that the women wanted a helping professional who would have empathy for them and that it was more difficult to relate to someone with a different background than themselves. Providing female clients with female mentors or therapists to enhance client-helping professional relatability is a common strategy for several existing treatment programs [30,31,32].

In addition to positive characteristics, the participants in this study also provided examples of negative characteristics of providers or programs. For example, high counselor turnover, misunderstandings about limits to confidentiality, feeling judged, feeling that the helping professional was not educated about sex and/or drugs, a lack of personal connection to the helping professional, feeling like they were forced into treatment, and not having a say in the treatment received were the main sources of the women’s negative prior experiences. The majority of the women talked about their own lack of participation in treatment as adolescents stemming from negative experiences with providers and programs.

These findings point to clear opportunities to increase client participation in treatment, specifically, the relatability and consistency of helping professionals. The women recommended that helping professionals be selected who are fun, kind, and show that they are listening and positive regardless of what the client shares. In addition, while there are myriad reasons for turnover in helping professionals, strategies such as improving the organizational climate by reducing stress [33,34] and increasing salaries [34] have been shown to reduce turnover.

**Specific content.** One of the more revealing aspects of the interviews was the lack of practical information the women had as adolescents, and their impressions of the lasting negative impact of this on their lives. Most of the women talked about specific and practical helpful things they remembered learning in prior programs such as healthy coping skills, emotion regulation, sex education, and the risks of drug use. They said that learning about accessing practical resources such as medication, food, shelter, education, money, and social support were invaluable to reducing their dependence on partners. In addition, the women reflected on the value of learning to participate in healthy, safe, and exciting activities (such as sports, art, and clubs). In fact, they stated that these activities directly replaced their time and desire to use drugs or alcohol. The women also talked about how they wished they had been taught more about the consequences of risky sexual behavior and drug use, how to talk to their partner about safe sex and drug use, and that it is okay to abstain from sex and/or drugs. They also wished they had been taught how to find practical resources (e.g., condoms, housing). For example, when they talked about wishing they had understood the risks of sex and drugs, they went into detail about specific risks on these issues (prevalence and consequences of STIs, side effects of pregnancy, financial burdens of having a child, and physical and emotional consequences of drugs use). They felt they needed to learn in hands-on ways, such as role-playing talks with partners and specifics on how a condom actually works and how to put it on. These findings point to the importance of pairing practical information with skills. This is consistent with a review of federal policies and programs showing that adolescents benefit from sex education that includes abstinence and harm-reduction strategies [35], and that information alone is not effective [36,37].

### 4.2. Limitations and Future Directions

This study was conducted in one Pacific Northwest city with limited racial diversity and had a sample of 15 women. Although findings provide insights into a topic that has not been extensively explored and has very tangible implications, findings may not be generalizable on a large scale. Due to the retrospective nature of this study, the women might not have remembered information that is important to understanding their experiences as adolescents. Future research could add to our findings in two specific ways. First, future studies should ask similar questions to more ethnically and gender diverse populations to see whether the current pattern of findings is generalizable. Learning the perspectives of men in similar circumstances would provide insight into how sex education and intervention programs can be implemented on a broad scale. Learning the perspectives of a more ethnically diverse population would also provide insight into how to implement these programs in more ethnically diverse cities. Second, future research could follow a similar population from childhood through adulthood to note changes over time. If adolescents were asked questions about their experiences while in treatment programs and then followed up with as young adults, researchers could gain valuable insight into how an adolescent’s opinion during adolescence is likely to change and develop as they age out of the program and enter adulthood. 

## 5. Conclusions

Results from the interviews consistently illustrated that the women in this study want adolescent girls with maltreatment histories to be given practical, reliable, and understandable information and skills. In addition, the women suggest that such information should be provided by a helping professional who the adolescent relates to and trusts. The women had many suggestions for ways in which helping professionals can build trust and be relatable to adolescent clients, such as maintaining confidentiality, being non-judgmental, allowing sufficient time to build rapport, and engaging them in positive, fun activities. The women’s stories suggest that adolescent girls armed with information and skills will feel more confident to not only respond effectively to social pressure, but also to think critically about the impact of risky behavior on their own lives. 
